# Real-world analysis of teclistamab in 123 RRMM patients from Germany

**DOI:** 10.1038/s41375-024-02154-5

**Published:** 2024-01-20

**Authors:** C. Riedhammer, F. Bassermann, B. Besemer, M. Bewarder, F. Brunner, A. Carpinteiro, H. Einsele, J. Faltin, J. Frenking, D. Gezer, S. Goldman-Mazur, M. Hänel, M. Hoegner, K. M. Kortuem, J. Krönke, M. Kull, T. Leitner, C. Mann, R. Mecklenbrauck, M. Merz, A. Morgner, A. Nogai, M. S. Raab, R. Teipel, R. Wäsch, L. Rasche

**Affiliations:** 1 Department of Internal Medicine II, University Hospital of Würzburg, Würzburg, Germany; 2 Department of Medicine III, Klinikum rechts der Isar, TUM, Munich, Germany; 3 Department of Hematology, Oncology, and Immunology, University Hospital of Tübingen, Tübingen, Germany; 4 Department of Hematology, Oncology, Clinical Immunology, Rheumatology, Saarland University Medical Center, Homburg, Germany; 5 Department of Internal Medicine IV, University Hospital of Halle, Halle, Germany; 6 Department of Hematology and Stem Cell Transplantation, University Hospital of Essen, Essen, Germany; 7 Department of Hematology and Stem Cell Transplantation, Helios-Klinik Berlin Buch, Berlin, Germany; 8 Heidelberg Myeloma Center, Department of Internal Medicine V, University Hospital of Heidelberg, Heidelberg, Germany; 9 Department of Hematology, Oncology, Hemostaseology and Stem Cell Transplantation, Faculty of Medicine, RWTH Aachen University, Aachen, Germany; 10 Center for Integrated Oncology Aachen Bonn Cologne Düsseldorf (CIO ABCD), Aachen, Germany; 11 Department of Hematology, Cell therapy and Hemostaseology, University Hospital of Leipzig, Leipzig, Germany; 12 Department of Internal Medicine III, Klinikum Chemnitz, Chemnitz, Germany; 13 Department of Hematology, Oncology and Cancer Immunology, Campus Benjamin Franklin, Charité-Universitätsmedizin Berlin, Berlin, Germany; 14 Department of Internal Medicine III, University Hospital of Ulm, Ulm, Germany; 15 Department of Hematology and Oncology, University Hospital Schleswig-Holstein, Campus Lübeck, Lübeck, Germany; 16 Department of Hematology, Oncology and Immunology, University Hospital of Gießen and Marburg, Marburg, Germany; 17 Department of Hematology, Hemostasis, Oncology and Stem Cell Transplantation Hannover Medical School, Hannover, Germany; 18 Department of Internal Medicine I, University Hospital of Dresden, Dresden, Germany; 19 Department of Medicine I, Medical Center - University of Freiburg, Faculty of Medicine, University of Freiburg, Freiburg, Germany

**Keywords:** Myeloma, Immunotherapy

## Abstract

Teclistamab, a B-cell maturation antigen (BCMA) × CD3 directed bispecific antibody, has shown high response rates and durable remissions in the MAJESTEC-1 trial in patients with relapsed and refractory multiple myeloma (RRMM). We retrospectively assessed efficacy and tolerability in 123 patients treated at 18 different German centers to determine whether outcome is comparable in the real-world setting. Most patients had triple-class (93%) or penta-drug (60%) refractory disease, 37% of patients had received BCMA-directed pretreatment including idecabtagene vicleucel (ide-cel) CAR-T cell therapy (21/123, 17.1%). With a follow-up of 5.5 months, we observed an overall response rate (ORR) of 59.3% and a median progression-free survival (PFS) of 8.7 months. In subgroup analyses, we found significantly lower ORR and median PFS in patients with extramedullary disease (37%/2.1 months), and/or an ISS of 3 (37%/1.3 months), and ide-cel pretreated patients (33%/1.8 months). Nonetheless, the duration of response in ide-cel pretreated patients was comparable to that of anti-BCMA naive patients. Infections and grade ≥3 cytopenias were the most frequent adverse events. In summary, we found that teclistamab exhibited a comparable efficacy and safety profile in the real-world setting as in the pivotal trial.

## Introduction

Bispecific antibodies and CAR-T cells are currently altering the therapeutic landscape of relapsed and refractory multiple myeloma (RRMM). In clinical trials, novel immune therapies have displayed high response rates, resulting in durable remissions lasting over a year [1–5].

With regard to CAR-T cell treatment with idecabtagene vicleucel (ide-cel) however, some discrepancies were observed in real-world conditions compared to the KarMMa trial with progression free survival times of 8.5 months vs. 12.1 months (optimal dose group) and overall survival times of 12.5 months vs. 19.4 months, respectively [2, 6]. This may be due to the trial´s strict entry criteria and generally observed benefits for patients treated in clinical trials, regardless of the treatment arm.

It is unclear whether such discrepancies also exist for teclistamab, a B-cell maturation antigen (BCMA) x CD3 bispecific antibody [7] which was approved for treatment of RRMM in several countries in 2022. In the MAJESTEC-1 trial, an overall response rate of 63% was observed with a median progression free survival of 11.3 months and median overall survival of 18.3 months [8]. Cytokine release syndrome and neurotoxicity appeared to be well manageable with grade 3 or 4 events in less than 1% of treated patients [8, 9]. Yet, the majority of patients experienced infections (76.4%) and therapy-induced neutropenia (70.9%) or other cytopenias [8]. Inclusion criteria of the MAJESTEC-1 trial comprised stable blood counts and kidney function [8]. We anticipated variations in the real-world patient cohort treated with teclistamab and sought to evaluate the effectiveness and tolerability in a representative group with access to both CAR-T and ADC therapy.

## Methods

This is an investigator-initiated retrospective study including 123 patients from 18 German centers who had received at least one full treatment dose of teclistamab between July 2022 and October 2023. Patient records were analyzed retrospectively. After step up doses of 0.06 and 0.3 mg/kg, teclistamab was applied weekly at doses of 1.5 mg/kg according to the label. Patients were evaluated retrospectively for meeting selected key inclusion criteria of the MAJESTEC-1 trial at screening for teclistamab (Supplementary Table 1). Outcomes were assessed according to the IMWG response criteria [10]. In addition, near complete remission was defined as serological complete remission lacking bone marrow assessment, as this was not always part of clinical routine. In patients with non-secretory disease, response evaluation was based on radiological criteria as previously described [10]. High-risk cytogenetic aberrations were defined as the presence of del(17p), t(4;14) and/or t(14;16).

Time-to-event analyses were conducted using the Kaplan–Meier method. For comparison of survival amongst subgroups, the log-rank test and Cox regression analysis were performed for univariable and multivariable analyses, respectively. Chi square tests were used to analyze differences in overall response rates between groups.

Adverse events such as hematologic toxicity and infections were graded according to Common Terminology Criteria for Adverse Events version 5.0 (https://ctep.cancer.gov/protocoldevelopment/electronic_applications/docs/ctcae_v5_quick_reference_5x7.pdf).

This retrospective study was approved by the local Ethics Committees (20230404 01 and 23-11299-BO).

## Results

### Patient characteristics

123 patients have received at least one full dose of teclistamab in 18 German centers from July 2022 to October 2023. Therapy had been discontinued during step-up dosing in another three other patients due to cytopenias, rapidly progressive disease or infections. Patient characteristics are shown in Table 1 in comparison to the patient cohort in the MAJESTEC-1 trial. Our cohort comprised higher proportions of patients with EMD, ISS of 3, high bone marrow infiltration and triple-class or penta-drug refractory disease. Most patients treated with teclistamab in our cohort had no remaining treatment options and many didn’t meet clinical trial eligibility criteria.

**Table 1:** Patient characteristics at baseline in comparison to MAJESTEC-1.

Characteristic	MAJESTEC-1	Real-world
Median age (range) - yr	64.0 (33.0–84.0)	67.0 (35.0–87.0)
Gender: male/female - %	58.2/41.8	56.9/43.1
Median time since diagnosis - yr (range)	6.0 (0.8–22.7)	6.5 (0.5–18.7)
Median no. of lines of previous therapy (range)	5 (2–14)	6 (3–14)
Extramedullary disease - no./total no. (%)	28/165 (17.0)	43/119 (36.1)
≥60% plasma cells in bone marrow no./total no. (%)	18/160 (11.2)	21/59 (35.6)
ISS no./total no. (%)		
I	85/162 (52.5)	25/92 (27.1)
II	57/162 (35.2)	35/92 (38.0)
III	20/162 (12.3)	31/92 (33.7)
High risk cytogenetic profile no./total no. (%)	38/148 (25.7)	39/106 (36.8)
Refractory status no./total no. (%)		
triple-class	128/162 (77.6)	113/123 (92.6)
penta-drug	50/162 (30.3	74/123 (60.2)

The patients were heavily pretreated with a median of 6 (range 3–14) prior lines of therapy. Of the 123 patients, 49 (39.8%) had undergone polychemotherapy regimens like PACE, including 22 patients (17.9%) with therapy within the last two months before teclistamab initiation. The vast majority (92.6%) had triple-class refractory disease and 60.2% of patients had penta-drug refractory disease. A substantial proportion of 39.0% would not have met the inclusion criteria of the MAJESTEC-1 trial shown in Supplementary Table 1. 37.4% (45/123) of the patients had received BCMA-directed pretreatment, among them 17.1% (21/123) with ide-cel, 18.7% (23/123) with belantamab mafodotin, and single patients with both or a BCMA-directed study medication. The median time between the last BCMA-directed treatment and the initiation of teclistamab was 6.0 months in anti-BCMA-pretreated patients.

### Efficacy

**Fig. 1:**
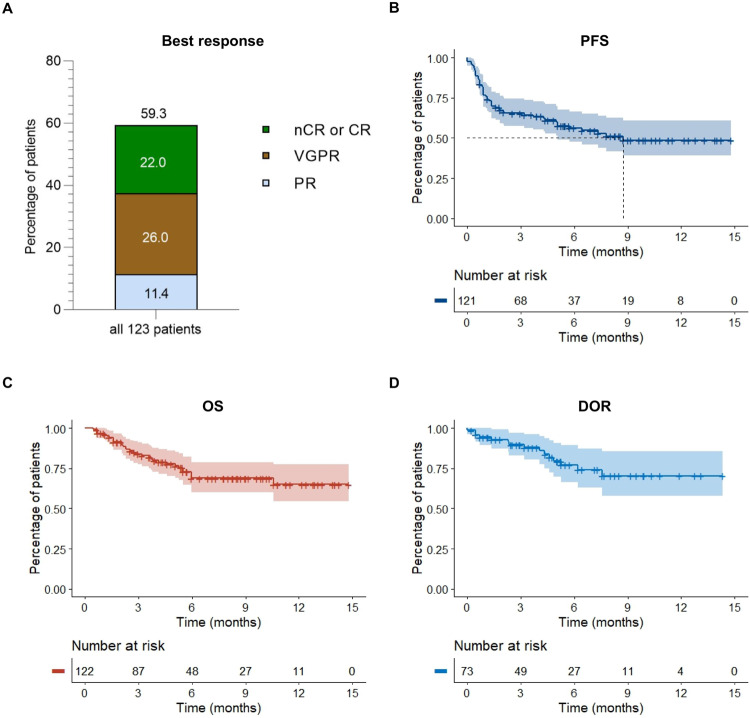
Rate of response in 123 patients and Kaplan–Meier analysis of response duration and of progression-free and overall survival. Panel **A** shows the rates of near complete response and complete response (CR), very good partial response (VGPR), and partial response in 123 patients who were treated with teclistamab. Panel **B** illustrates progression-free survival and Panel **C** overall survival among the 123 patients. Panel **D** shows the duration of response to teclistamab therapy in the 73 patients who had an overall response (partial response or better). Tick marks indicate censored data. Bands indicate confidence bands around survival curves.

In our real-world study, 59.3% of patients responded to treatment with teclistamab achieving partial remission or better (Fig. 1). 22.0% reached a complete or near complete response, 26.0% had a very good partial remission and 11.4% partial remission. 13.0% did not respond but maintained stable disease, 25.2% showed primary progressive disease. Median time to response was 1.0 months, median time to best response 1.6 months. With a median follow-up of 5.5 months, median progression free survival (PFS) time was 8.7 months (with still 55% censored events at data cut-off). Median duration of response (DOR) and median overall survival (OS) times were not reached (Fig. 1).

### Efficacy in BCMA pretreated patients and high-risk subgroups

**Fig. 2:**
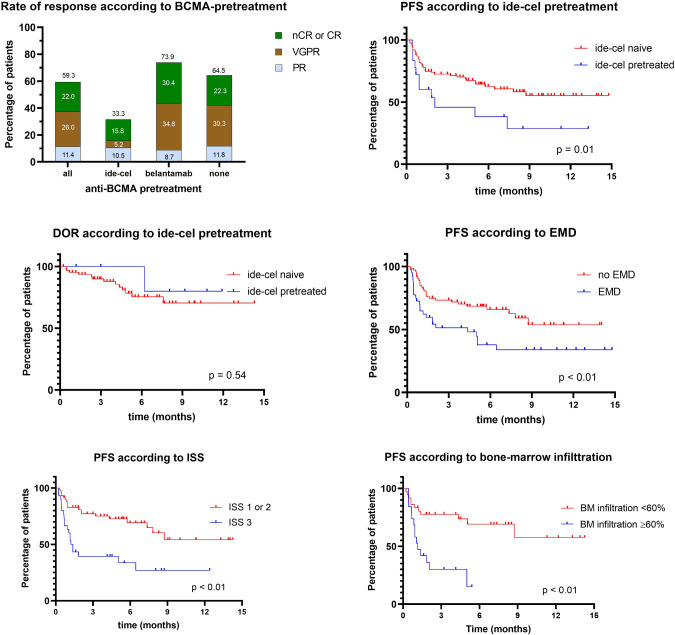
Response to teclistamab in subgroups. Rate of response according to BCMA-pretreatment, PFS and DOR in ide-cel pretreatment and PFS in further subgroups (EMD, ISS 3, bone marrow infiltration ≥60%).

The ORR for patients with BCMA-directed pretreatment was lower at 54.8% compared to anti-BCMA naive patients with an ORR of 64.5%. Notably, this disparity was exclusively attributable to patients pretreated with ide-cel (*n* = 21) who had an ORR of only 33.3% (*p* < 0.01, chi-square test) (Fig. 2). In contrast, the ORR for patients with belantamab pretreatment (73.9%) was comparable to the ORR in anti-BCMA naive patients (64.5%) (Fig. 2). Patients pretreated with ide-cel exhibited a significantly lower median PFS of 1.8 months (*p* = 0.01, log-rank test). However, the DOR was not reached in patients with PR or better and did not differ from that of patients naive to ide-cel (*p* = 0.54, log-rank test) (Fig. 2). At data cutoff, six out of seven responding patients with ide-cel pretreatment remained in remission. Interestingly, among the four long-term responders (benefiting for >8 months), one patient had demonstrated primary refractory disease to ide-cel, and the other three patients all experienced early relapse (<180 days). Three of the 21 patients had undergone ide-cel pretreatment only shortly (<1.5 months) before teclistamab initiation, two of them achieving VGPR with teclistamab treatment. Response rates to teclistamab did not differ between patients with an interval between CAR-T cell treatment and teclistamab initiation of more or less than 3 months (33% vs. 20%, *p* = 0.57, chi-square test) or 6 months (22% vs. 36%, *p* = 0.49, chi-square test), respectively.

In further subgroup analyses, patients with EMD and an ISS of 3 at teclistamab initiation showed a significantly inferior ORR and progression free survival time in univariable and multivariable analysis (see Tables 2 and 3 and Fig. 2). Bone marrow infiltration ≥60% was not included in multivariable analysis due to limited case numbers, but showed significantly inferior PFS and ORR in univariable analysis. In contrast, there was no significant difference in ORR and PFS time between patients with penta-refractory disease compared to those without penta-refractory disease and between patients who received polychemotherapy within the last two months compared to those who did not. Furthermore, ORR and PFS did not significantly differ among patients with different cytogenetic risk profiles (see Tables 2 and 3 and Supplementary Fig. 1). Of note, PFS of patients treated with ide-cel showed a trend towards inferior outcomes in the multivariable analysis, albeit the limited case numbers (*p* = 0.07, *n* = 21).

**Table 2:** Univariable and multivariable models of the association of selected patient characteristics with PFS.

PFS in univariable and multivariable analysis						
Characteristic	*n* (event *n*)	Median PFS in months	Univariable		Multivariable	
			HR (95% CI)	*p*	HR (95% CI)	*p*
EMD				<0.01		<0.01
No	73 (26)	NR	1.00 (referent)		1.00 (referent)	
Yes	43 (24)	2.07	2.31 (1.29–4.14)		3.00 (1.63–5.51)	
ISS 3				<0.01		<0.01
No	50 (22)	NR	1.00 (referent)		1.00 (referent)	
Yes	31 (20)	1.30	2.47 (1.25–4.88)		2.46 (1.32–4.58)	
Bone marrow infiltration ≥ 60%				<0.01		
No	38 (11)	NR	1.00 (referent)		Not performed	Not performed
Yes	21 (14)	1.05	3.58 (1.56–8.24)		Not performed	Not performed
High-risk cytogenetics				0.36		
No	67 (22)	NR	1.00 (referent)		Not performed	Not performed
Yes	39 (16)	8.74	1.33 (0.70–2.55)		Not performed	Not performed
Ide-cel pretreatment				<0.01		0.07
No	102 (37)	NR	1.00 (referent)		1.00 (referent)	
Yes	21 (11)	1.84	2.56 (1.31–5.83)		1.87 (0.95–3.71)	
Penta-refractoriness				0.39		
No	49 (18)	NR	1.00 (referent)		Not performed	Not performed
Yes	74 (32)	7.34	1.28 (0.74–2.21)		Not performed	Not performed
Polychemotherapy in last 2 months				0.10		
No	101 (39)	NR	1.00 (referent)		Not performed	Not performed
Yes	22 (11)	3.22	1.67 (0.80–3.46)		Not performed	Not performed

**Table 3:** Association of selected patient characteristics with the overall response rate.

ORR in univariable analysis				
Characteristic	*n* (event *n*)	Response rate (%)	OR (95% CI)	*p*
EMD				<0.01
No	75 53)	72.6	1.00 (referent)	
Yes	43 (16)	37.2	4.47 (2.00–9.99)	
ISS 3				<0.01
No	61 (41)	67.2	1.00 (referent)	
Yes	30 (11)	36.7	3.54 (1.39–9.13)	
Bone marrow infiltration ≥ 60%				<0.01
No	36 (24)	66.7	1.00 (referent)	
Yes	21 (5)	23.8	6.40 (1.93–19.8)	
High-risk cytogenetics				0.96
No	66 (38)	57.6	1.00 (referent)	
Yes	38 (22)	59.0	0.98 (0.46–2.23)	
Ide-cel pretreatment				<0.01
No	99 (66)	66.7	1.00 (referent)	
Yes	21 (7)	33.3	4.00 (1.52–10.1)	
Penta-refractoriness				0.49
No	48 (31)	64.6	1.00 (referent)	
Yes	72 (42)	58.3	1.30 (0.63–2.83)	
Polychemotherapy in last 2 months				0.25
No	98 (62)	63.3	1.00 (referent)	
Yes	22 (11)	50.0	1.72 (0.67–4.41)	

### Safety

Safety outcomes were comparable to those observed in the MAJESTEC-1 trial. During step-up dosing, 58.5% of patients developed CRS and 7.3% neurotoxic events in the form of immune-effector cell-associated neurotoxicity syndrome (ICANS). Tocilizumab was administered in 23.6% and dexamethasone in 16.2% of patients. Like in the MAJESTEC-1 trial, grade ≥3 events for CRS and ICANS were rare (Table 4). The median hospital stay for step-up dosing was 10 days. Seven patients (5.7%) required intensive care unit support for management of CRS or ICANS, infections or other complications. Frequent adverse events observed included infections and cytopenias. Any kind of infection occurred in 54.5% of the patients, 49.3% of them (26.8% of all patients) requiring hospitalization for grade ≥3 infections. In all centers, patients treated with teclistamab received continuous PJP and HSV prophylaxis. IVIG substitution was used as primary and/or secondary infection prophylaxis with different approaches in different participating centers. Additionally, a majority (53.7%) of patients experienced grade ≥3 cytopenias of any kind according to CTCAE (Table 4). G-CSF and TPO agonizts were used in 22.0% and 3.3% of patients, respectively. Treatment interruptions of more than two and four weeks occurred in 33.1% and 19.8% of patients, respectively.

**Table 4:** Adverse events in 123 patients.

Adverse event	Any grade	Grade ≥ 3
Infections no. (%)	67/123 (54.5)	33/123 (26.8)
CRS no. (%)	72/123 (58.5)	2/123 (1.6)
ICANS no. (%)	9/123 (7.3)	1/123 (0.8)
Cytopenia any kind no. (%)	110/123 (89.4)	66/123 (53.6)
Neutropenia no. (%)	68/123 (55.3)	46/123 (38.0)
Anemia no. (%)	99/123 (80.5)	39/123 (31.7)
Thrombopenia no. (%)	78/123 (63.4)	32/132 (26.0)

## Discussion

In this real-world analysis across multiple centers, we observed outcomes in 123 RRMM patients that were similar to those seen in the pivotal trial. For instance, the ORR of 64.5% in our BCMA-naive group was nearly equal to the ORR of 63% in MAJESTEC-1 [8]. It is noteworthy that almost half of our patients did not meet the key inclusion criteria of the clinical trial. PFS was slightly lower at 8.7 months (vs. 11.3 months in MAJESTEC-1), but follow-up was limited to a median of 5.5 months and 55% of the data were censored at the time of data cut. Lower CR rates in our real-world analysis (22% vs. 39% in the MAJESTEC-1 trial) can also be attributed to a shorter median follow-up time, as responses have been shown to deepen over time. We also observed markedly poorer outcomes among patients with EMD, an ISS of 3, and/or ≥60% bone marrow infiltration. While patient characteristics such as age, gender, and lines of pretreatment remained consistent with the pivotal trial, our real-world group was enriched by high risk features such as ISS 3, high risk cytogenetic aberrations, EMD, or high bone marrow infiltration. The off-the-shelf availability of teclistamab likely contributed to the similar outcomes in the pivotal trial and our real-world analysis. The ability to initiate treatment quickly in rapidly evolving disease settings is a major benefit of bispecific antibodies compared to CAR-T cell therapy.

Both our real-world analysis and the MAJESTEC-1 trial demonstrated decreased efficacy of teclistamab in patients with EMD. EMD is an established risk factor [11] and seems to hold its negative prognostic impact in the era of T-cell based immunotherapy [3, 12, 13]. In line with this assumption, Zanwar et al. recently reported a median PFS of only 2.9 months in patients with EMD treated with bispecific antibodies [14]. Furthermore, we found a high tumor load to be associated with an inferior outcome. Lower efficacy of bispecific antibodies in these settings has been previously reported in preclinical models of BCMA x CD3 bispecific antibodies [15] and is well described for the CD19 x CD3 bispecific antibody blinatumomab in acute lymphoblastic leukemia [16–18]. Likewise, the CD3 x BCMA-directed bispecific antibody elranatamab showed lower efficacy in patients with EMD and an ISS of 3 [4, 19]. The mechanisms of impaired efficacy of bispecific antibodies in patients with high tumor burden and EMD remain incompletely understood. Altered conditions that hinder the entry of specific T-cells into the tumor lesions may play a role in EMD [20] as well as an increased degree of T-cell exhaustion in patients with abundant tumor cell counts [16, 21].

A key question in this context is whether debulking chemotherapy can enhance response in patients with high tumor load. 23 patients in our cohort had received polychemotherapy such as PACE in the two months before teclistamab treatment. The median PFS of this group was not significantly different from that of other patients, considering the limited number of cases. At the same time, chemotherapy may affect T-cell fitness as described for ide-cel [22] or CD19 bispecifics [23].

Interestingly, our study and the MAJESTEC-1 trial revealed no differences in outcomes between patients with high-risk cytogenetics and those without. Even patients with two or more high-risk cytogenetic aberrations, categorized as having “ultra high-risk disease”, for whom other therapies have reported significantly inferior outcomes [24], did not exhibit differences in response rates or PFS time in our study. However, the presence of high-risk cytogenetics is likely to be underreported in our real-world cohort, as bone marrow punctures including cytogenetic analyses are not always part of clinical routine.

Another clinically relevant observation was the efficacy of teclistamab in patients previously treated with anti-BCMA therapies. Initial reports already described lower response rates to teclistamab treatment after BCMA-directed CAR-T cell therapies [25]. Our study also observed lower response rates and PFS in patients pretreated with ide-cel. However, the duration of response in patients achieving a PR or better (7/21) was similar to ide-cel naive patients. Therefore, we believe that teclistamab continues to be a valuable treatment option for patients pretreated with ide-cel - a setting with limited therapeutic options. BCMA-loss, as previously described [26–30] may be one potential mechanism for primary resistance to teclistamab after BCMA therapy, although drivers of resistance may be heterogeneous [31]. Interestingly, all four patients with long-term remissions following teclistamab experienced only limited benefit to ide-cel treatment. In these patients, CAR-T product-intrinsic issues including insufficient T-cell expansion may play a role. Future efforts to pre-identify non-responders will be important to avoid futile and costly BCMA-directed retreatment, and antigen expression testing could help in this setting. However, the frequency of (functional) BCMA loss has yet to be determined and a standard approach to testing for these aberrations is missing. In contrast, patients treated with Belantamab did not exhibit distinct outcomes. In patients enrolled in the DREAMM-1 and DREAMM-2 trials, BCMA loss was not reported [32], thus reinforcing the idea that the pressure on the clonal architecture is lower with antibody drug conjugates.

Safety was comparable to that observed in MAJESTEC-1. Neurotoxicity and cytokine release syndrome appeared to be low-grade in the majority of cases and well manageable. However, infections were frequent and posed a significant challenge in the treatment with teclistamab. The infection rate of 54.4% in this real-world analysis was lower than that reported in the MAJESTEC-1 trial, likely due to the shorter follow-up in our study [8]. The high rates of infections require close monitoring and adequate preventive measures [33, 34].

In conclusion, teclistamab displays a similar safety and efficacy profile to that in the MAJESTEC-1 trial and is a valuable treatment option for RRMM. Further studies are warranted to evaluate a potential role of teclistamab in less advanced treatment lines or newly diagnosed MM.

### Supplementary information

supplementary material

## Data Availability

The datasets generated during and/or analyzed during the current study are available from the corresponding author on reasonable request

## References

[1] Topp MS, Duell J, Zugmaier G, Attal M, Moreau P, Langer C (2020). Anti-B-cell maturation antigen BiTE molecule AMG 420 induces responses in multiple myeloma. J Clin Oncol.

[2] Munshi NC, Anderson LD, Shah N, Madduri D, Berdeja J, Lonial S (2021). Idecabtagene vicleucel in relapsed and refractory multiple myeloma. N Engl J Med.

[3] Martin T, Usmani SZ, Berdeja JG, Agha M, Cohen AD, Hari P (2023). Ciltacabtagene autoleucel, an anti-B-cell maturation antigen chimeric antigen receptor T-cell therapy, for relapsed/refractory multiple myeloma: CARTITUDE-1 2-year follow-up. J Clin Oncol.

[4] Lesokhin AM, Tomasson MH, Arnulf B, Bahlis NJ, Miles Prince H, Niesvizky R (2023). Elranatamab in relapsed or refractory multiple myeloma: phase 2 MagnetisMM-3 trial results. Nat Med.

[5] Rasche L, Hudecek M, Einsele H (2020). What is the future of immunotherapy in multiple myeloma?. Blood.

[6] Hansen DK, Sidana S, Peres LC, Colin Leitzinger C, Shune L, Shrewsbury A (2023). Idecabtagene vicleucel for relapsed/refractory multiple myeloma: real-world experience from the myeloma CAR T Consortium. J Clin Oncol.

[7] Pillarisetti K, Powers G, Luistro L, Babich A, Baldwin E, Li Y (2020). Teclistamab is an active T cell-redirecting bispecific antibody against B-cell maturation antigen for multiple myeloma. Blood Adv.

[8] Moreau P, Garfall AL, van de Donk NWCJ, Nahi H, San-Miguel JF, Oriol A (2022). Teclistamab in relapsed or refractory multiple myeloma. N Engl J Med.

[9] Usmani SZ, Garfall AL, van de Donk NWCJ, Nahi H, San-Miguel JF, Oriol A (2021). Teclistamab, a B-cell maturation antigen × CD3 bispecific antibody, in patients with relapsed or refractory multiple myeloma (MajesTEC-1): a multicentre, open-label, single-arm, phase 1 study. Lancet.

[10] Kumar S, Paiva B, Anderson KC, Durie B, Landgren O, Moreau P (2016). International Myeloma Working Group consensus criteria for response and minimal residual disease assessment in multiple myeloma. Lancet Oncol.

[11] Bladé J, Beksac M, Caers J, Jurczyszyn A, von Lilienfeld-Toal M, Moreau P (2022). Extramedullary disease in multiple myeloma: a systematic literature review. Blood Cancer J.

[12] Chari A, Minnema MC, Berdeja JG, Oriol A, van de Donk NWCJ, Rodríguez-Otero P (2022). Talquetamab, a T-cell-redirecting GPRC5D bispecific antibody for multiple myeloma. N Engl J Med.

[13] Wang Y, Cao J, Gu W, Shi M, Lan J, Yan Z (2022). Long-term follow-up of combination of B-cell maturation antigen and CD19 chimeric antigen receptor T cells in multiple myeloma. J Clin Oncol.

[14] Zanwar S, Ho M, Lin Y, Kapoor P, Binder M, Buadi FK (2023). Natural history, predictors of development of extramedullary disease, and treatment outcomes for patients with extramedullary multiple myeloma. Am J Hematol.

[15] Meermeier EW, Welsh SJ, Sharik ME, Du MT, Garbitt VM, Riggs DL (2021). Tumor burden limits bispecific antibody efficacy through T-cell exhaustion averted by concurrent cytotoxic therapy. Blood Cancer Discov.

[16] Duell J, Dittrich M, Bedke T, Mueller T, Eisele F, Rosenwald A (2017). Frequency of regulatory T cells determines the outcome of the T-cell-engaging antibody blinatumomab in patients with B-precursor ALL. Leukemia.

[17] Wei AH, Ribera J-M, Larson RA, Ritchie D, Ghobadi A, Chen Y (2021). Biomarkers associated with blinatumomab outcomes in acute lymphoblastic leukemia. Leukemia.

[18] Topp MS, Gökbuget N, Stein AS, Zugmaier G, O’Brien S, Bargou RC (2015). Safety and activity of blinatumomab for adult patients with relapsed or refractory B-precursor acute lymphoblastic leukaemia: a multicentre, single-arm, phase 2 study. Lancet Oncol.

[19] Bahlis NJ, Costello CL, Raje NS, Levy MY, Dholaria B, Solh M (2023). Elranatamab in relapsed or refractory multiple myeloma: the MagnetisMM-1 phase 1 trial. Nat Med.

[20] Robinson MH, Villa NY, Jaye DL, Nooka AK, Duffy A, McCachren SS, et al. Regulation of antigen-specific T cell infiltration and spatial architecture in multiple myeloma and premalignancy. J Clin Invest. 2023;133, 10.1172/JCI167629. 10.1172/JCI167629 PMC10378152 37526080

[21] Chow A, Perica K, Klebanoff CA, Wolchok JD (2022). Clinical implications of T cell exhaustion for cancer immunotherapy. Nat Rev Clin Oncol.

[22] Rytlewski J, Madduri D, Fuller J, Campbell TB, Mashadi-Hossein A, Thompson EG (2020). Effects of prior alkylating therapies on preinfusion patient characteristics and starting material for CAR T cell product manufacturing in late-line multiple myeloma. Blood.

[23] Duell J, Lukic DS, Karg M, Reusch U, Koch J, Zhukovsky EA (2019). Functionally defective T cells after chemotherapy of B-cell malignancies can be activated by the tetravalent bispecific CD19/CD3 antibody AFM11. J Immunother.

[24] Callander N, Silbermann R, Kaufman JL, Godby KN, Laubach JP, Schmidt TM (2022). Analysis of transplant-eligible patients (pts) who received frontline daratumumab (DARA)-based quadruplet therapy for the treatment of newly diagnosed multiple myeloma (NDMM) with high-risk cytogenetic abnormalities (HRCA) in the Griffin and master studies. Blood.

[25] Touzeau C, Krishnan AY, Moreau P, Perrot A, Usmani SZ, Manier S (2022). Efficacy and safety of teclistamab (tec), a B-cell maturation antigen (BCMA) x CD3 bispecific antibody, in patients (pts) with relapsed/refractory multiple myeloma (RRMM) after exposure to other BCMA-targeted agents. J Clin Oncol.

[26] Lee H, Ahn S, Maity R, Leblay N, Ziccheddu B, Truger M (2023). Mechanisms of antigen escape from BCMA- or GPRC5D-targeted immunotherapies in multiple myeloma. Nat Med.

[27] Da Vià MC, Dietrich O, Truger M, Arampatzi P, Duell J, Heidemeier A (2021). Homozygous BCMA gene deletion in response to anti-BCMA CAR T cells in a patient with multiple myeloma. Nat Med.

[28] Samur MK, Fulciniti M, Aktas Samur A, Bazarbachi AH, Tai Y-T, Prabhala R (2021). Biallelic loss of BCMA as a resistance mechanism to CAR T cell therapy in a patient with multiple myeloma. Nat Commun.

[29] Leblay N, Maity R, Barakat E, McCulloch S, Duggan P, Jimenez-Zepeda V (2020). Cite-seq profiling of T cells in multiple myeloma patients undergoing BCMA targeting CAR-T or bites immunotherapy. Blood.

[30] Samur MK, Martin N, Thompson E, Fulciniti M, Aktas-Samur A, Kaiser S (2022). Differences in single cells between BCMA-targeting CAR T-cell therapy responders and non-responders reveals initial resistance and acquired resistance are driven by different factors. Blood.

[31] Rasche L, Vago L, Mutis T. Tumour escape from CAR-T cells. In: Kröger N, Gribben J, Chabannon C, Yakoub-Agha I, Einsele H editors. The EBMT/EHA CAR-T Cell Handbook, Cham: Springer; 2022. 15–22. 36122074

[32] Lowther DE, Houseman EA, Han G, Kleanthous E, Knoblock D, Zhou X (2022). No evidence of BCMA expression loss or systemic immune impairment after treatment with the BCMA-targeted antibody-drug conjugate (ADC) belantamab mafodotin (Belamaf) in the DREAMM-1 and DREAMM-2 trials of patients with relapsed/refractory multiple myeloma (RRMM). Blood.

[33] MM-192 preliminary recommendations for prevention and management of infections, hypogammaglobulinemia, and neutropenia during treatment with teclistamab based on experience from the MajesTEC-1 study. Clin Lymphoma Myeloma Leuk. 2023;23:S480–S481.

[34] Raje N, Anderson K, Einsele H, Efebera Y, Gay F, Hammond SP (2023). Monitoring, prophylaxis, and treatment of infections in patients with MM receiving bispecific antibody therapy: consensus recommendations from an expert panel. Blood Cancer J.

