# Supplementary material for: Real-world analysis of teclistamab in 123 RRMM patients from Germany
Source: Leukemia. 2024 Jan 20;38(2):365–71. doi: 10.1038/s41375-024-02154-5 (PMC10844072; doi:10.1038/s41375-024-02154-5)
Supplement: Supplementary file 1 — supplementary material [file 41375_2024_2154_MOESM1_ESM.docx]

**Supplementary Table 1.**

| **Selection of inclusion criteria in MAJESTEC-1** |
| --- |
| Measurable disease (M-protein level >1.0 g/dl/urine M-protein level >200 mg/24h or FLC >10 mg/dl and abnormal FLC ratio) |
| Platelets ≥75 x 10^9^/l for pat. in whom <50% of bone marrow nucleated cells are plasma cells; otherwise platelet count ≥50 x 10^9^/l |
| Absolute neutrophil count (ANC) ≥1.0 x 10^9^/l |
| Creatinine clearance ≥40 ml/min/1.73m^2^ |
| no allogeneic transplantation |
| no known active CNS involvement |
| no plasma cell leukemia |

**Supplementary Figure 1. PFS in different cytogenetic risk profiles**


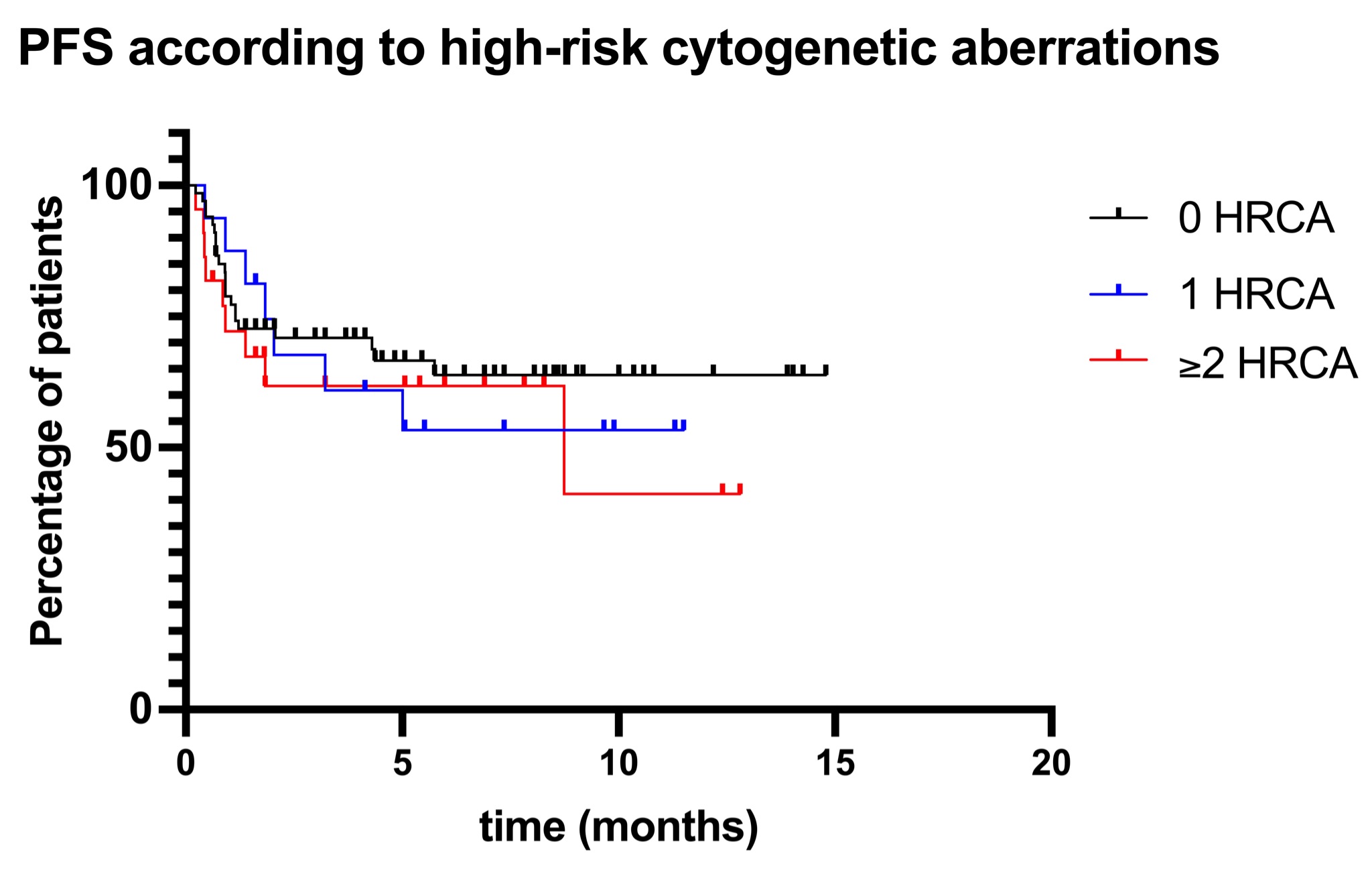


PFS in patients with 0, 1 or ≥2 high-risk cytogenetic aberrations (HRCA) is shown. Patients with 1 HRCA had either del(17p), t(4;14), or t(14;16). In patients with 2 HRCA, two aberrations were present, including del(17p), t(4;14), t(14;16) and gain/amp1q.
